# Disordered Patterns in Clustered Protein Data Bank and in Eukaryotic and Bacterial Proteomes

**DOI:** 10.1371/journal.pone.0027142

**Published:** 2011-11-04

**Authors:** Michail Yu. Lobanov, Oxana V. Galzitskaya

**Affiliations:** Group of Bioinformatics, Institute of Protein Research Russian Academy of Sciences, Pushchino, Moscow Region, Russia; University College Dublin, Ireland

## Abstract

We have constructed the clustered Protein Data Bank and obtained clusters of chains of different identity inside each cluster, http://bioinfo.protres.ru/st_pdb/. We have compiled the largest database of disordered patterns (141) from the clustered PDB where identity between chains inside of a cluster is larger or equal to 75% (version of 28 June 2010) by using simple rules of selection. The results of these analyses would help to further our understanding of the physicochemical and structural determinants of intrinsically disordered regions that serve as molecular recognition elements. We have analyzed the occurrence of the selected patterns in 97 eukaryotic and in 26 bacterial proteomes. The disordered patterns appear more often in eukaryotic than in bacterial proteomes. The matrix of correlation coefficients between numbers of proteins where a disordered pattern from the library of 141 disordered patterns appears at least once in 9 kingdoms of eukaryota and 5 phyla of bacteria have been calculated. As a rule, the correlation coefficients are higher inside of the considered kingdom than between them. The patterns with the frequent occurrence in proteomes have low complexity (PPPPP, GGGGG, EEEED, HHHH, KKKKK, SSTSS, QQQQQP), and the type of patterns vary across different proteomes, http://bioinfo.protres.ru/fp/search_new_pattern.html.

## Introduction

Intrinsically disordered regions serve as molecular recognition elements, and play an important role in the control of many cellular processes and signaling pathways [1]–[6]. It is useful to be able to predict positions of disordered regions in protein chains. Prediction methods are aimed at identifying disordered regions through the analysis of amino acid sequences using mainly the physicochemical properties of amino acids [7]–[16] or evolutionary conservation [17]–[20].

Many examples of proteins with intrinsically disordered regions which exhibit coupling between folding and binding have been described in the literature [4]–[6], [21]–[23]. Nevertheless, the universality of this phenomenon and functional importance of many disordered regions remain unclear.

A database of continuous protein fragments (Molecular Recognition Features or MORFs) was compiled from the Protein Data Bank which includes short protein chains (with fewer than 70 residues) bound to larger proteins [24], [25]. It has been argued that MORFs participate in the coupling of binding and folding, a hypothesis that was supported by the analysis of the composition and predicted disorder of MORF segments. As a result of studying the subtle structural differences of the same proteins in bound (Complex) and unbound (Single) states in relation to their intrinsic disorder the database of protein structures (ComSin) has been constructed [26].

Recently several computational tools for identifying Linear motifs [27] and minimotifs in protein-protein interactions [28] have been published. Linear motifs are short segments of multidomain proteins that provide regulatory functions independently of protein tertiary structure [27] but minimotifs are short functional peptide sequences obtained after analysis of known protein-protein interactions [28].

Low-complexity regions attract our attention since they are regions of a protein in which a particular amino acid, or a small number of different amino acids, are enriched. Single amino acid repeats (homorepeats) belong to these regions. It turned out that homorepeats play important roles in some biological process [29] and may play a more important role in human diseases than it was previously recognized.

In the current study we search for sequence patterns consisting of a number of consecutive residues along the polypeptide chain that are nearly always associated with disordered segments. It has been found that two types of patterns appear to be recurrent: a proline-rich pattern and a positively or negatively charged pattern [30]. It should be noted that the old and new versions of our libraries include patterns enriched by proline and charged residues [31].

The statistical analysis of disordered residues was done considering 34 464 unique protein chains taken from the PDB database. In this database, 4.95% of residues are disordered (i.e. invisible in X-ray structures) [31]. The statistics was obtained separately for the *N*- and *C*-termini as well as for the central part of the protein chain. It has been shown that frequencies of occurrence of disordered residues of 20 types at the termini of protein chains differ from the ones in the middle part of the protein chain [31], [32].

It is necessary to construct a clustered PDB because this simplifies the filtering process of protein structures under their analysis and searches general structural characteristics among non-identical proteins. It is necessary to construct a clustered PDB which is important for the analysis of actualized data.

In this work we constructed a clustered PDB and used clusters of protein chains where identity between chains inside of the cluster exceeds 75% (version of 28 June 2010). Combining the motif discovery and disorder protein segment identification in the clustered PDB allows us to create the largest library of disordered patterns [31]. At present the library includes 141 disordered patterns. Such an approach is new and promising for further studying and understanding the functional role of the obtained patterns in different proteomes. Taking into consideration the library of disordered patterns will help one improve accuracies of predictions for residues to be structured or unstructured inside the given region. The previous version of the library includes 109 disordered patterns and has restrictions on the minimal length of the patterns. Using more simple rules without restriction on the pattern length and clustered PDB of the same version we constructed the largest library of disordered patterns.

The patterns occur more often as short fragments. Patterns of four-six residues long occur more frequently (105 out of 141) among the disordered patterns of the library. It should be noted that six residue patches affect the folding/aggregation features of proteins, and they are important “words” for the understanding of protein dynamics [33]. Moreover, nucleation sites are constrained by patches of approximately six residues [34], [35]. There is evidence that the minimum length necessary for a peptide to elicit an allergenic response and molecular mimicry (a patch of a protein eliciting an immune response equivalent to the entire protein) is about six [36]. All these facts suggest the existence of a fragment of biologically meaningful information located along approximately six residues [33].

With the library of disordered patterns taken into account, it would be easier to improve accuracy of prediction of ordered/disordered residues inside the given region.

Proteome-wide calculations are a great way to place our work in a larger, evolutionary frame. In this paper of interest is the occurrence of 141 disordered patterns in 97 eukaryotic proteomes, since eukaryotic proteomes include more disordered regions than other proteomes [17], [37], [38], and for comparison, in 26 bacterial proteomes. A comparative analysis of the number of proteins containing the 141 disordered selected patterns in these proteomes has been performed. The disordered patterns with the most frequent occurrence in eukaryotic and bacterial proteomes have low complexity.

It should be noted that each proteome has a specific set of disordered patterns, and this results in different correlation coefficients between numbers of proteins where a disordered pattern appears at least one time. We came to some important observations of a higher correlation coefficient within a kingdom or a phylum than across kingdoms or phyla after analysis of occurrence of disordered patterns in 123 proteomes. The disordered patterns appear more often in eukaryotic than in bacterial proteomes. One can suggest that such short similar motifs are responsible for common functions for nonhomologous, unrelated proteins from different organisms.

## Materials and Methods

### Construction of clustered PDB

We have considered all protein structures determined by X-ray analysis with a resolution better than 3 Å, and the size of protein is larger than or equal to 40 amino acid residues, published in the PDB (version of June 28, 2010); the structures contain 116 997 protein chains (51 048 PDB entries). At the first step these 116 997 chains can be divided into 34 464 classes. We call these classes as clusters with 100% identity. This means that the chains from the same cluster have the same amino acid sequences, the sequences of chains from different classes are different i.e. they differ at least at one position. In total these 34 464 different sequences contain 9 085 893 residues. At the second step we created clusters of chains with identity inside each cluster ≥75%.

Identity is calculated by using equation:
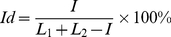
(1)where *I* is the number of identical residues, *L_1_* and *L_2_* are the numbers of amino acid residues in each considered protein. For calculation of Identity we used BLAST with default parameters [39].

**Figure 1:**
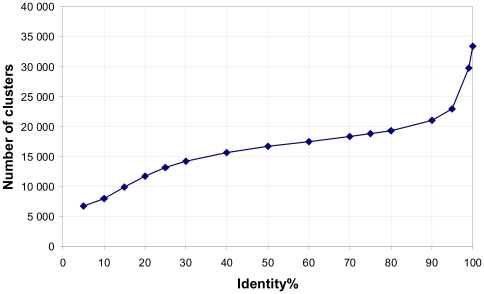
Dependence of the number of clusters on identity between protein chains. Inside each cluster at the given identity between chains the identity is larger than the considered identity between clusters.

At the beginning a pair of chains with maximal identity was combined, then another pair of chains or a chain with the cluster again with maximal identity, etc. If the combining of a chain with the cluster or combining of clusters occurred, then the average identity of gathering was considered. If identity of at least a pair of chains from different clusters was less than 75%, then the clusters were not combined. The procedure was repeated until there were clusters which could be combined. At the second step of grouping of chains, we obtained 18775 clusters of chains with identity inside each cluster ≥75%. Then the clusters C75 have been combined into clusters with identity *Id*≥50%, etc. Figure 1 demonstrates the dependence of the number of clusters on identity between chains inside the cluster. Further we consider the identity of 75% because the general grouping has occurred below 90% identity.

### Construction of the library of disordered patterns

Among 116 997 chains, approximately 4.5% of their residues are disordered, i.e. are not resolved by X-ray analysis. To reveal such residues, we compared (for each protein chain) records SEQRES and records ATOM in the corresponding PDB-file. Residues which were present in record SEQRES, but their coordinates were absent in record ATOM (namely, the coordinates of the C_α_-atom were absent in record ATOM), were considered as unstructured ones. We considered the residues as disordered if there were not coordinates of C_α_ atoms.

**Figure 2:**
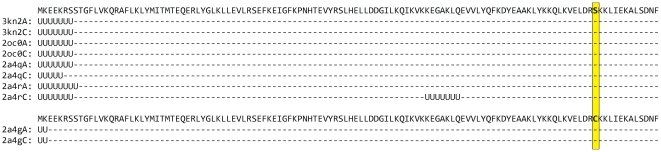
Example of two clusters with 100% identity combined in one cluster with 75% identity. The sequences from two clusters are different only in one position (110), serin is changed for cystein. U denotes disordered residues in the chain and dash denotes ordered residues, respectively.

Below we consider only clusters with ≥75% identities between any pair of chains inside each cluster because the general grouping has occurred below 90% identity. Considering this level of identity, we have created the Clustered Disordered Residues Data Base (CDRDB), its elements are 18 775 clusters of protein chains. Figure 2 illustrates two clusters with 100% identity combined in one cluster with 75% identity. One can see that the sequences from two clusters are different in one position 110, serin is changed for cystein, and the weight of the chain from the first cluster is
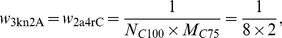
(2)and the weight of the chain from the second cluster is 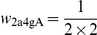, respectively. Analogously the weight of each chain from any cluster is calculated by using equation:
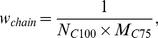
(3)where *N_C100_* is the number of chains in the cluster with 100% identity and *M_C75_* is the number of clusters with 75% identity. It should be noted that the sum of weights inside one cluster with 75% identity will be equal to one. The weight of residue we consider to be the same as the weight of chain so at the level of 75% identity a cluster may include protein chains of different lengths.

Our goal is to create a database of disordered patterns i.e. amino acid sequences that are likely to be found in disordered parts of protein chains using CDRDB by applying simpler rules for the creation of the library of disordered patterns than in our previous work [31]. Let *P* be a protein chain and *A* be a pattern of length *L*. The database was compiled using a two-stage procedure. At the first stage, we created a list of candidate patterns. To be a candidate in the patterns the considered pattern should be disordered in half cases among the chains from the cluster with 100% identity. Then the desired disordered patterns were selected into the candidate list. 855 775 candidates in the disordered patterns were gathered.

We say that pattern *A* matches chain *P* at position *s* if the following conditions are valid:

1. two residues from each end should coincide:
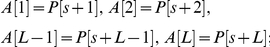

2. there could be done substitutions at most *L/5* positions *r* in the middle of pattern in which
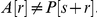

This means that for patterns with a length of L≤5 no change is possible, for 5<L≤10 – only 1 change, for 10<L≤15 – 2 changes, etc. The occurrence is terminal if it belongs to the first 40 residues (“*N*-terminal”) or last 40 residues (“*C*-terminal”) of the chain. The other occurrences are called internal ones.

If the distance between the edges of the pattern and the chain is less than 40 residues the pattern is considered to match these residues. *The pattern length is not limited in this paper*. Further we consider the following terminology: N_u_ is the sum of weights (*w_chain_*) of disordered residues matched by the pattern; N_f_ is the sum of weights (*w_chain_*) of ordered residues matched by the pattern; C_u_ is the number of clusters with identity 75% (C75) in which N_u_>N_f_; C_f_ is the number of clusters with identity 75% (C75) in which N_u_≤N_f_. Protein *P* has an occurrence of pattern *A* if *A* matches *P* at position *s*.

Fragment *A = P_j_[s+1, s+L]* of chain *P_j_* is considered as a candidate disordered pattern if it meets the following conditions:
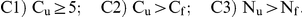



There are 16 918 patterns meeting conditions C1, C2, and C3. The longest pattern has the length of 45 amino acid residues (HHHHHHSSGLVPRGSGMKETAAAKFERQHMDSPDLGTDDDDKAMA), and the shortest pattern has 2 residues (HH). In the next step we selected disordered patterns from the candidate list using the following iterative greedy procedure. From 16 918 patterns we chose the pattern with the maximal value D = N_u_−N_f_. Then for the rest patterns the values of N_u_, N_f_, C_u_, C_f_ were recalculated not taking into account the residues matched by the first pattern. Again all the rest patterns were checked to meet conditions C1, C2, and C3. Among the rest patterns meeting conditions C1, C2, and C3 the pattern with a maximal D value was chosen. If there were no patterns meeting conditions C1, C2, and C3, then the procedure was stopped. The iterative procedure was stopped when 390 patterns were selected (D>0). Finally, we were interested in the patterns for which D≥10 and D≥25 (the value 25 corresponds to the summation of weights of 5 whole disordered patterns with 5 residues in length in 5 clusters without neighboring regions, or terminal occurrence). The numbers of such patterns are 249 and 141, respectively (see Dataset S1). The lengths of patterns are in the region: 4≤L≤24. Further we will consider only the set of patterns meeting the condition that D≥25.

### Significance of disordered occurrences

We have studied the statistical significance of the selected patterns from two points of view. First, we were interested whether the disordered fragments are overrepresented among the occurrences of each pattern, and, second, whether the patterns are overrepresented in the database. The features are described with the proper Z-scores, called *Z_disorder_* and *Z_occur_*, respectively. To estimate the significance of the number of disordered occurrences of pattern *P* we have implemented the following procedure. First, we determined the fraction of disordered fragments among all fragments with the given length taking into account the weight of the disordered residues in each case:
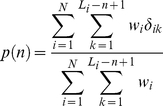
(4)where *N* is the number of chains in the CDRDB, *L_i_* is the length of the considered chain, *n* is the fragment length, *w_i_* is the chain weight, 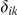 is equal to 1 if the fragment with adjoining regions is disordered more than in half positions, and 0 in the opposite case. For each pattern we know the number of clusters C_u_ where this pattern in more than half cases is disordered, and also the number of clusters C_f_ where this pattern is folded in more than half cases (see Dataset S1, columns J and K). We should calculate the probability *P* (Y) that the number of successes will be larger or equal to C_u_ at the given number of trials Y = C_u_+C_f_.

In other words, this is the probability that at the given or larger number of trials:
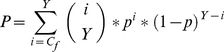
(5)where *p* is the probability of success of one trial (see above). The significance of disordered occurrences is estimated with the *Z*-score:
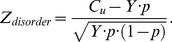
(6)


### Statistical significance of the observed number of occurrences of pattern *X* in proteomes

The probability of finding patterns with possible changes is equal to the summation of probabilities over all sequences compatible with the given pattern.
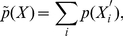
(7)

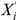
 is the sequence compatible with the given one (see the rules of coincidence, for example i = 39 for n = 6).
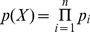
(8)where the probability *p(X)* that pattern *X* occurs in a sequence and *p_i_* is the probabilities of occurrence of amino acids in the considered proteome. We calculated the probability *p(X, N)* that pattern *X* with *n* amino acid residues occurs in a sequence of length *N*:
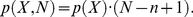
(9)


The probability distribution on protein sequences is assumed to be binomial.

The statistical significance of pattern *X* is estimated with the Z-score
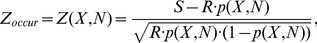
(10)where *S* is the number of sequences containing at least one occurrence of homorepeat *X. R* is the number of proteins in the considered proteome. *N* is the average length of proteins in the considered proteome.

### Statistical significance of the observed number of occurrences of pattern *X* in two different proteomes

Let *n_i_* and *n_j_* be the numbers of proteins with the given pattern X in proteomes *i* and *j*. *N_i_* and *N_j_* are the whole numbers of proteins in both proteomes, and 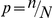 is the frequency of proteins with the given pattern. 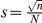 is the standard deviation. *L_i_* and *L_j_* are the average length of proteins in the considered proteomes *i* and *j*. The scoring function is:
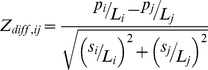
(11)We consider that the difference is significant if its Z-score exceeds the proper value with absolute meaning 3 and 5. These values correspond to the probabilities 3*10^−3^ and 6*10^−7^, respectively.

The correlation coefficient (r) was calculated using the equation:
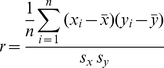
(12)where *S_x_* and *S_y_* are standard deviations for variables *x* and *y*.

### Database of proteomes

We considered 3279 proteomes from the EBI site (ftp://ftp.ebi.ac.uk/pub/databases/SPproteomes/uniprot/proteomes/). Since the patterns with the frequent occurrence in proteomes have low complexity we did a preliminary analysis. The analysis showed that the number of proteins with at least one occurrence of homorepeats of 6 residues long is less than 500 for proteomes with an overall number of residues below 2500000. Even so, only 22 proteomes out of 3156 have more than 100 proteins with at least one occurrence of 6-residue homorepeats. The data gave grounds for our research involving only proteomes with an overall number of residues exceeding 2500000.

We obtained 123 proteomes taking into account the length of proteomes representing 9 kingdoms of eukaryotes and 5 phyla of bacteria (see Table 1 and Dataset S2). Unfortunately, only three kingdoms of eukaryotes (Metazoa, Viridiplantae, and Fungi) are given at http://www.ncbi.nlm.nih.gov/Taxonomy/. In other cases, the rank of kingdom is missing. In such situations, we chose the highest taxonomic category proceeding from the subkingdom of eukaryotes instead of the kingdom. We chose 97 out of 120 eukaryotic proteomes, and a small number of bacterial proteomes. The smallest eukaryotic proteome belongs to *Hemiselmis andersenii*, class Cryptophyta. It is evident that 498 proteins with an overall number of 167452 of amino acid residues are not sufficient for reliable statistics. Historically, the superkingdom of bacteria is divided into phyla but not kingdoms. We preferred to consider such phyla separately.

**Table 1:** Names of 97 eukaryotic and 26 bacterial proteomes.

Eukaryota		Eukaryota (Fungi)	Bacteria***	
Metazoa	25.H_sapiens	34310.A_capsulata_ATCC_26029	Acidobacteria	25797.S_usitatus
	22974.B_taurus	34967.A_capsulata_H143	Actinobacteria	37022.A_mediterranei
	59.M_musculus	34495.A_dermatitidis_SLH14081		33926.C_acidiphila
	122.R_norvegicus	34498.A_dermatitidis_ER-3		35278.Frankia_sp_EuI1c
	21457.G_gallus	35919.A_benhamiae		35534.F_sp
	20721.D_rerio	29154.A_clavatus		74443.K_setae
	22388.T_nigroviridis	33020.A_flavus		33113.R_opacus
	17.D_melanogaster	22118.A_fumigatus_FGSC_A1100		25456.Rhodococcus_sp
	25396.D_pseudoobscura	31018.A_fumigatus_CEA10		131.S_avermitilis
	31436.A_aegypti	29130.A_niger		36666.S_bingchenggensis
	78607.A_darlingi	23077.A_oryzae		84.S_coelicolor
	22426.A_gambiae	28239.A_terreus		34910.S_scabies
	21633.C_briggsae	30100.B_fuckeliana		35554.S_sp_ACT-1
	9.C_elegans	22024.C_albicans_SC5314		58962.S_violaceusniger
	64800.L_loa	32738.C_dubliniensis		34011.S_roseum
	79720.T_spiralis	19665.C_glabrata	Proteobacteria	112.B_japonicum
	30565.N_vectensis	34491.C_tropicalis		22343.Burkholderia_sp_ATCC_17760
Viridiplantae	23214.O_sativa	25585.C_globosum_IFO_6347		25388.B_xenovorans
	3.A_thaliana	34493.C_lusitaniae		33223.H_ochraceum
	33157.Micromonas_sp	34218.C_posadasii		23351.M_xanthus
	29351.O_lucimarinus	79902.C_graminicola		32044.P_pacifica
	25972.O_tauri	20018.D_hansenii		30295.S_cellulosum
Stramenopiles*	35109.E_siliculosus	34482.L_thermotolerans		33616.S_aurantiaca
Choanoflagellida**	30562.M_brevicollis	29447.L_elongisporus	Bacteroidetes	33930.C_pinensis
Euglenozoa*	83400.L_braziliensis	22028.M_oryzae		32144.M_marina
	83363.L_infantum	34471.N_otae	Chloroflexi	36622.K_racemifer
	71330.T_brucei	34970.N_haematococca		
	33602.T_cruzi	29157.N_fischeri		
Alveolata*	32114.P_berghei	22025.N_crassa		
	31998.P_chabaudi	34307.P_brasiliensis_Pb03		
	493.P_falciparum	34389.P_brasiliensis_Pb18		
	31342.P_knowlesi	34392.P_brasiliensis_ATCC_MYA-826		
	31632.P_vivax	31898.P_chrysogenum		
	21631.P_yoelii	32999.P_marneffei		
Amoebozoa*	21395.D_discoideum	25591.P_nodorum		
	35301.P_pallidum	29448.P_guilliermondii		
Diplomonadida*	33600.G_intestinalis_ATCC_50803	28727.P_stipitis		
	35295.G_intestinalis_ATCC_50581	79908.P_graminis		
	65115.G_intestinalis	79905.P_teres		
		30091.S_cerevisiae_YJM789		
		31651.S_cerevisiae_RM11-1a		
		34506.S_cerevisiae_JAY291		
		35062.S_cerevisiae_Lalvin_EC1118		
		71242.S_cerevisiae		
		30103.S_sclerotiorum		
		35280.S_macrospora		
		33056.T_stipitatus		
		35921.T_verrucosum		
		34386.U_reesii		
		30097.V_polyspora		
		35359.V_albo-atrum		
		20011.Y_lipolytica		
		31020.C_cinerea		
		20846.C_neoformans_JEC21		
		21380.C_neoformans_B-3501A		
		31023.L_bicolor		
		33031.P_placenta		
		22029.U_maydis		

*Category without rank is given.

**The name of order is given because the highest ranks are missing in the taxonomic description.

***The superkingdom of bacteria is divided in phyla rather than kingdoms.

Among 97 eukaryotic proteomes, 17 belong to the kingdom of Metazoa or animals: *Homo sapiens* (51778 protein sequences), *Bos Taurus* (18405), *Mus musculus* (42120), *Rattus norvegicus* (28166), *Gallus gallus* (12954), *Danio rerio* (21576), and *Tetraodon nigroviridis* (27836) belong to Chordata phylum, *Drosophila melanogaster* (15101), *Drosophila pseudoobscura* (16000), *Aedes aegypti* (16042), *Anopheles darlingi* (11437), and *Anopheles gambiae* (12455) to arthropods, and *Caenorhabditis briggsae* (18531), *Caenorhabditis elegans* (23817), Loa loa (16271), and *Trichinella spiralis* (16040) belong to nematodes, *Nematostella vectensis* (24435) belongs to cnidaria phylum.

## Results and Discussion

### Library of disordered patterns

**Figure 3:**
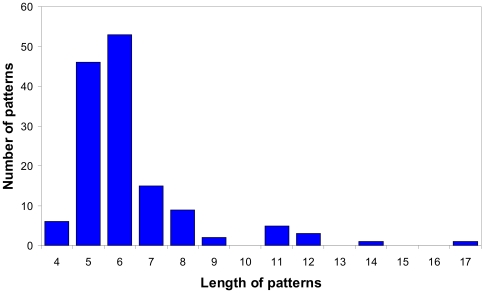
Dependence of the number of patterns on the length (number of amino acid residues).

Following the procedure described in the Materials and Methods section, we constructed the clustered PDB (CDRDB) at the level identity of 75% (http://bioinfo.protres.ru/st_pdb/) and obtained a library of disordered patterns. The dataset includes 141 patterns (see Dataset S1). Figure 3 demonstrates the distribution of the patterns according to their lengths. The patterns occur more frequently as short fragments (105 out of 141 are patterns of 4–6 residues long). The largest pattern with condition D≥25 consists of 17 amino acid residues (HHHHHHSSGLEVLFQGP). It is interesting that the strong pattern is HHHH, but not HHHHHH as in the last version of the library [31]. We suggest that the residues matched by these patterns will be disordered in new protein chains because more than half of residues in these patterns are disordered (see conditions C2 and C3 in the Materials and Methods section).

The statistical significance of disordered occurrences in the selected patterns was estimated with the *Z-*score (see Materials and Methods). We calculated the probability that the number of successes will be larger or equal to C_u_ at the given number of C_u_+C_f_ (for each pattern we know the number of clusters C_u_ where this pattern in more than half cases is disordered, and also the number of clusters C_f_ where this pattern is ordered in more than half cases). This probability for all 141 disordered patterns is less than 7•10^−5^.

All 141 patterns have *Z_disorder_>6.4* that corresponds to the P-value of 7•10^−5^, which is in good agreement with the procedure of the disordered patterns determination. The worst variant is C_u_ = 5, C_f_ = 4, and the length of patterns is 6. We have four such cases: SVAESS, ASIGQA, PPSGSP, and DSDVSL (see Dataset S1, columns O and P).

### Comparison of the new and the previous libraries of disordered patterns

After construction of the new library the question about similarity of two databases (previous and new) arises. For this purpose the previous patterns matched the clustered pdb (CDRDB) and the sum of weights was calculated analogously to the new patterns. Then we calculated the sum of weights for residues matching both the previous and the new patterns (intersections, I_12_). The number of clusters with identity of 75% in which there were new and previous patterns was calculated, as well as the number of intersections. The degree of coincidence was calculated using equations (13) and (14):
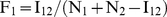
(13)

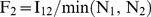
(14)where I_12_ is the sum of weights for intersections (coincidences), and N is the weight of a single pattern. We considered only pairs where F_2_>0.1, F_2_(C75)>0.1, I_12_≥3, and the number of clusters where two patterns appear together, C_12_≥3 (see Dataset S3). The measure F_1_ points to the coincidence between two considered patterns. At the same time the measure F_2_ demonstrates a level of inclusion of the pattern with smaller N into a larger one. Large difference between N_1_ and N_2_ results in a wide difference between F_1_ and F_2_.

For example, the sequence GSSHH**HHHH**SSGLVPR**GSHM** occurs in 393 clusters on the N-termini, where it is disordered more than half in 387 clusters. This sequence is matched by pattern GSHM, and its beginning is matched by the HHHH pattern. If we have a test database with one protein where there is such a sequence at the N-end, then N_GSHM_ = 20. N is the weight of a pure pattern with the neighboring part, in this case this is the length of the whole N-terminal fragment, N_HHHH_ = 9, I_12_ = 9, F_1_ = 9/20 = 0.45, F_2_ = 9/9 = 1. In a real situation in the whole CDRDB N_HHHH_ = 29 560.4, N_GSHM_ = 8 452.0, I_12_ = 3 163.1, F_1_ = 0.09, F_2_ = 0.37. It should be noted that real F_2_ is less than test F_2_. This occurs because GSHM appears usually in sequence GSS**HHHHHH**SSGLVPR**GSHM** or in similar sequences. Yet sometimes GSHM appears alone.

**Figure 4:**
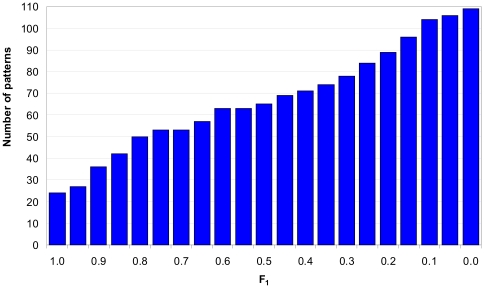
Dependence of the number of coinciding patterns between previous and new libraries of disordered patterns at the given level of coincidence (F_1_). The measure F_1_ points to the coincidence of protein regions covered by the considered patterns.

The result of intersections of the two libraries (the previous library includes 109 patterns and the new one includes 390 patterns if D>0) is presented in Fig. 4. One can see that there are 16 precise coinciding patterns: ENLYFQ, ASMTGGQQMGR, GSSHHH, WSHPQFEK, EGGSHHHHH, RRGKKK, PTTENLYFQGAM, PTTENLYFQGAM, SHHHHHHSQDP, HHHHHMA, SMTGGQQMGRGS, KKGEKK, SRSHHHH, ENLYFGGS, GGRHHH, HHHGSM, GSHMSQ, and 8 with not precise coincidence, for example HHHHHH and HHHH (Dataset S3).

**Figure 5:**
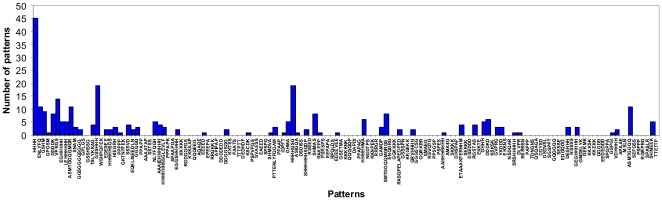
Number of patterns with which the given pattern appears together in the same protein in the clustered PDB. Pattern HHHH appears 45 times together with some other patterns from the library and 84 patterns appear alone in the clustered PDB.

It is interesting that some patterns appear in a protein together with other patterns (57 out of 141). Such pairs can be seen in Dataset S4. Also we calculated the number of patterns which appear in proteins together with the considered pattern (see Fig. 5). Pattern HHHH occurs more often with other patterns in proteins. It should be noted that there are several patterns which appear alone in the CDRDB (see Fig. 5, Dataset S4). We used the same criteria as for the intersections of the two libraries.

### Occurrence of disordered patterns in 97 eukaryotic and 26 bacterial proteomes

After creating the library of disordered patterns taken from the CDRDB, another interesting question arises: how often the obtained patterns could occur in some proteomes. Since eukaryotic proteomes include more disordered regions than other proteomes [17], [37], [38] we compared 97 eukaryotic proteomes and 26 bacterial ones (see Table 1, Dataset S1, and Materials and Methods).

We considered two cases for coincidence. In the first case we calculated the number of proteins where the patterns match with precise coincidence a polypeptide chain fragment. In the second case we analyzed the coincidence according to the definition suggested here and in the paper [31]. According to the rule mentioned in the Materials and Methods section for patterns with a length of L≤5 no change may occur, for 5<L≤10 – only 1 change may take place, for 10<L≤15 – 2 changes, etc.

**Figure 6:**
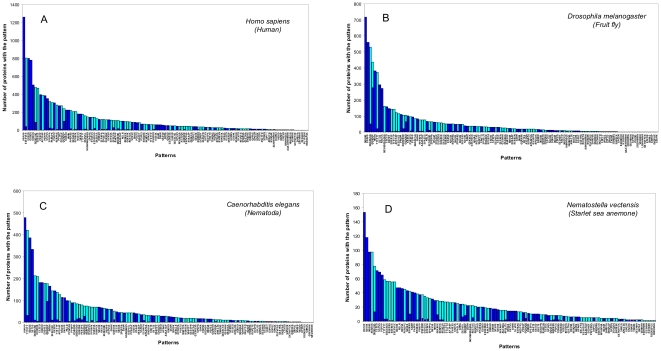
Occurrence of disordered patterns in four proteomes. (**A**) *H. sapiens*, Chordata phylum; (**B**) *D. Melanogaster*, Arthropoda phylum; (**C**) *C. elegans*, Nematoda phylum; (**D**) *N. vectensis*, Cnidaria phylum. The blue color corresponds to precise coincidence of the considered patterns with the fragment of polypeptide chains, the aqua color corresponds to exact coincidence of two terminal residues from both termini and incomplete coincidence in the L/5 positions.

Among 141 disordered patterns 17 occur (with precise coincidence) only in the PDB but are very sparse in 123 proteomes (see Dataset S5). Such patterns as RASQPELAPEDPED, SMTGGQQMGRGS, SHHHHHHSQDP, PTTENLYFQGAM, HHHHHHSSGLEVLFQGP, EQKLISEEDLN, and ASMTGGQQMGR do not appear in the analyzed proteomes even in two cases (precise coincidence and exact coincidence of two terminal residues and no coincidence in L/5 positions) (see Figure 6). This suggests that such patterns are an artificial addition to proteins from the CDRDB for their better purifications.

From Figure 6 it is evident that the homorepeats occur very often in eukaryotic proteomes. The patterns with the most frequent occurrence in the eukaryotic proteomes have low complexity: PPPPP, GGGGG, EEEED, HHHH, KKKKK, SSTSS, and QQQQQP. From Tables 2 and 3 it is evident that the disordered patterns with the most frequent occurrence in the eukaryotic and even in bacterial proteomes are patterns with low complexity GGGGG, PPPPP, TTTPTT, GGGGSGG, KKKKK, etc.

**Table 2:** Number of proteins with the most frequent occurrence of disordered patterns in 17 considered animal proteomes in the case of incomplete coincidence.

Number of proteins	51778	18405	42120	28166	12954	21576	27836	15101	16000	16042	11437	12455	18531	23817	16271	16040	24435
Proteome	*H. sapiens*	*B. taurus*	*M. musculus*	*R. norvegicus*	*G. gallus*	*D. rerio*	*T. nigroviridis*	*D. melanogaster*	*D. pseudoobscura*	*A. aegypti*	*A. darlingi*	*A. gambiae*	*C. briggsae*	*C. elegans*	*L. loa,16271*	*T. spiralis*	*N. vectensis*
PPPPP	1260	467	1002	739	282	352	581	560	526	276	385	326	318	478	127	130	153
GGGGG	779	339	606	435	198	270	522	718	885	391	1025	782	201	385	64	115	97
GGGGSGG	478	203	364	266	128	148	276	529	579	225	698	513	103	212	46	59	56
EEEED	800	320	604	524	235	340	350	159	170	140	182	186	215	182	41	67	71
EEEEVEE	804	279	650	581	189	295	346	114	112	68	130	125	148	144	49	54	55
HHHH	224	71	201	138	67	164	192	383	416	325	817	409	116	165	62	107	29
TTTPTT	178	56	160	110	45	102	76	373	412	256	579	283	271	420	232	199	58
KKKKK	503	330	423	353	179	564	196	102	88	139	84	91	169	181	78	79	118
SSTSS	353	115	310	218	125	181	237	295	277	218	301	168	238	333	116	82	69
QQQQQP	240	60	195	145	57	78	107	437	528	230	501	239	109	176	56	82	23
EDEREE	467	185	360	354	129	235	242	143	135	97	140	109	138	179	37	39	55
NSSSS	182	74	179	136	82	116	102	272	293	181	287	143	73	114	58	99	31
PAPPP	385	146	298	230	74	112	181	148	144	74	108	81	100	113	23	56	40
PPAPP	397	161	327	260	103	104	176	111	115	56	81	88	91	90	23	22	37
APIPAP	388	150	282	242	59	95	131	122	211	63	82	71	81	127	12	13	26
PSRSPS	310	113	251	225	79	129	204	98	111	66	72	54	59	79	31	18	45
KKGEKK	210	131	197	177	100	170	121	57	47	76	50	56	151	209	35	40	97
DDDDEDD	108	41	120	104	30	144	73	105	172	104	311	102	60	85	131	61	77
PSPPP	306	113	269	183	74	109	170	95	82	62	80	59	48	61	22	18	65
KKEKK	225	74	189	145	76	147	73	48	52	66	39	46	117	143	35	24	47

**Table 3:** Average number of proteins with the most frequent occurrence of disordered patterns in 123 considered proteomes in the case of incomplete coincidence.

	Metazoa (17)	Viridiplantae (5)	Stramenopiles (1)	Choanoflagellida (1)	Euglenozoa (4)	Alveolata (6)	Amoebozoa (2)	Diplomonadida (3)	Fungi (58)	Acidobacteria (1)	Actinobacteria (14)	Proteobacteria (8)	Bacteroidetes (2)	Chloroflexi (1)
GGGGG	460	1176	2691	75	277	41	409	7	121	75	67	73	21	26
PPPPP	468	679	618	133	297	18	249	25	171	54	38	70	14	24
TTTPTT	224	147	115	196	275	18	1826	17	112	10	23	10	8	19
GGGGSGG	287	562	1941	47	105	30	233	4	72	49	40	53	9	16
KKKKK	216	152	119	73	57	741	196	9	68	0	0	4	17	4
EEEED	270	233	348	71	107	103	280	7	98	1	1	3	6	10
SSTSS	214	118	135	96	104	28	455	53	106	2	7	9	5	18
EEEEVEE	244	187	470	53	125	94	299	8	83	0	1	4	3	10
QQQQQP	192	81	142	67	101	5	693	11	81	7	6	4	3	8
EDEREE	179	140	163	78	74	82	167	9	82	1	6	7	8	10
DDDDEDD	108	206	203	154	58	82	308	3	83	0	2	10	3	3
HHHH	229	168	99	44	58	17	292	1	62	4	4	5	5	10
APIPAP	127	174	179	116	43	3	30	19	63	42	84	88	5	22
PSRSPS	114	135	90	52	80	8	134	21	81	7	26	13	1	6
NSSSS	142	90	73	30	51	54	534	18	53	2	2	4	3	8
PAPPP	136	155	89	59	55	4	27	6	53	39	33	44	5	8
PSPPP	107	236	133	48	85	5	60	8	56	8	10	15	0	6
PPAPP	132	134	102	50	87	2	21	4	41	17	35	44	4	10
KKGEKK	113	77	64	26	36	238	106	10	39	2	1	3	23	7
RGRPRG	89	161	132	55	41	11	17	3	44	4	21	18	2	10

According to work [31] we suggest that these patterns will be disordered in most cases. It should be noted that low-complexity regions can additionally include ordered structural proteins or proteins with strong structural propensity, like collagens, coiled-coils or fibrous proteins [40]. Recently, it has been demonstrated that an increased number of perfect tandem repeats correlates with their stronger tendency to be unstructured [41]. Moreover, strong association between homorepeats and unstructured regions was shown elsewhere [42]. Such patterns as GGGGSGG, EEEEVEE, EDEREE, APIPAP, and PSRSPS (see Table 2) often occur in the considered 17 animal proteomes.

It should be noted that poly H fragments are artificial parts of proteins in the PDB which have been added for better purification of proteins, but in eukaryotic proteomes such a repeat is likely to have a biological function. The locations of poly-H fragments can be found in different proteomes from our site, http://bioinfo.protres.ru/fp/search_new_pattern.html.

We calculated the statistical significance of the observed patterns in 123 proteomes by using equation (10) (see Materials and Methods). It should be noted that the average length of proteins in considered proteomes is larger (about 400 residues) than the average length of the protein in the PDB database (about 260 residues). On the one hand, Z_occur_≤0, varies from 40 patterns for the human proteome to 91 ones for the bacterial proteome *B. xenovorans*. On the other hand, Z_occur_>5 varies from 65 patterns in the rice proteome (*O. sativa*) to 8 patterns in the bacterial proteome *B. xenovorans*. Several examples deserve our attention. For instance, the appearance of pattern GGSGGGGSGGG varies from 7 cases in *T. spiralis* (the expected occurrence is 0.0004) to 149 cases in humans (the expected occurrence is 0.013), but the Z_occur_ value is 353 and 1291, respectively. Such patterns as MSLN and SNAM appear more sparsely in comparison with the expected value (Z_occur_<0) for all considered 17 animal proteomes. Although the first pattern occurs 100 times (that is not rare) in the human proteome, and the second pattern appears 61 times, correspondingly. At the same time pattern HHHH appears more often than expected (from 10 for the human to 4 for the actinia (*N.vectensis*)), but Z is 68 and 12, respectively.

We calculated the frequencies of occurrence of 141 disordered patterns in 123 proteomes. To make a statement that the given pattern X occurs more often in the *i* proteome than in the *j* one we introduced the scoring function for such difference between occurrences of the pattern in two proteomes (by using equation (11), see Materials and Methods). This scoring function should have a normal distribution according to the central limit theorem. We considered the difference occurrence of 141 patterns in some pairs of proteomes (see Dataset S5) and illustrated here the example for eukaryota and bacteria superkingdoms. It turns out that the appearances of 55 patterns in the two superkingdoms do not differ significantly at the level of 10^−7^. The negative value of the scoring function points out that the frequency of appearance of the given pattern is higher in bacteria than in eukaryota superkingdoms. For example pattern APIPAP occurs 1.5 times more frequently in 26 bacterial proteomes than in 97 eukaryotic proteomes (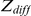 = −20.4). It should be added, that HHHH and QQQQQP patterns occur in Arthropoda's proteomes more often than in the Chordata proteomes (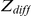 = −38.4 and −34.7, correspondingly) (see Table 2 and Dataset S5).

For each proteome we calculated a set of 141 values reflecting the number of proteins containing at least one disordered pattern for each of the 141 patterns from the library. Then considering all possible pairs of proteomes, the correlation coefficients between the 141 values have been calculated resulting in the matrix of correlation coefficients. The correlation coefficient was calculated for each pair of proteomes separately (see Table 4), and then averaging has been done inside each kingdom and phylum (see Table 5). As a rule, the correlation coefficients are higher inside the studied kingdom and phylum than between them.

**Table 4:** Correlation coefficients (in percent) between 17 animal proteomes (kingdom Metazoa).

Phylum	Proteome	*H. sapiens*	*B. taurus*	*M. musculus*	*R. norvegicus*	*G. gallus*	*D. rerio*	*T. nigroviridis*	*D. melanogaster*	*D. pseudoobscura*	*A. aegypti*	*A. darlingi*	*A. gambiae*	*C. briggsae*	*C. elegans*	*L. loa*	*T. spiralis*	*N. vectensis*
Chordata	*H. sapiens*		**98**	**99**	**99**	**97**	**86**	**97**	73	69	71	57	68	**84**	**80**	56	66	**83**
	*B. taurus*	**98**		**98**	**98**	**97**	**91**	**95**	71	67	70	56	67	**83**	**79**	54	65	**86**
	*M. musculus*	**99**	**98**		**99**	**97**	**88**	**97**	74	70	73	59	68	**86**	**82**	59	69	**85**
	*R. norvegicus*	**99**	**98**	**99**		**97**	**89**	**96**	69	65	70	54	64	**85**	**79**	57	66	**84**
	*G. gallus*	**97**	**97**	**97**	**97**		**92**	**95**	72	68	75	58	68	**88**	**83**	60	70	**88**
	*D. rerio*	**86**	**91**	**88**	**89**	**92**		**83**	62	58	70	53	59	**84**	**76**	63	70	**88**
	*T. nigroviridis*	**97**	**95**	**97**	**96**	**95**	**83**		**80**	**77**	**78**	67	**77**	**84**	**82**	57	69	**83**
Arthropoda	*D. melanogaster*	73	71	74	69	72	62	**80**		**99**	**95**	**94**	**96**	**79**	**87**	69	**83**	**67**
	*D. pseudoobscura*	69	67	70	65	68	58	**77**	**99**		**94**	**96**	**96**	**74**	**83**	66	**80**	62
	*A. aegypti*	71	70	73	70	75	70	**78**	**95**	**94**		**94**	**92**	**85**	**90**	**78**	**90**	73
	*A. darlingi*	57	56	59	54	58	53	67	**94**	**96**	**94**		**97**	68	**77**	67	80	54
	*A. gambiae*	68	67	68	64	68	59	77	**96**	**96**	**92**	**97**		71	**79**	60	**75**	61
Nematoda	*C. briggsae*	**84**	**83**	**86**	**85**	**88**	**84**	**84**	**79**	74	**85**	68	71		**97**	**84**	**88**	**86**
	*C. elegans*	**80**	**79**	**82**	**79**	**83**	**76**	**82**	**87**	**83**	**90**	**77**	**79**	**97**		**85**	**90**	**84**
	*L. loa*	56	54	59	57	60	63	57	69	66	78	67	60	**84**	**85**		**91**	74
	*T. spiralis*	66	65	69	66	70	70	69	**83**	**80**	**90**	**80**	**75**	**88**	**90**	**91**		74
Cnidaria	*N. vectensis*	**83**	**86**	**85**	**84**	**88**	**88**	**83**	67	62	73	54	61	**86**	**84**	74	74	

**Table 5:** Averaged correlation coefficients (in percent) between numbers of proteins where at least once a disordered pattern for each of 141 patterns appears in 9 kingdoms of eukaryota and 5 phyla of bacteria.

	Metazoa (17)	Viridiplantae (5)	Stramenopiles (1)	Choanoflagellida (1)	Euglenozoa (4)	Alveolata (6)	Amoebozoa (2)	Diplomonadida (3)	Fungi (58)	Acidobacteria (1)	Actinobacteria (14)	Proteobacteria (8)	Bacteroidetes (2)	Chloroflexi (1)
Metazoa (17)	**78**	71	59	70	**75**	32	55	47	**75**	55	43	48	52	69
Viridiplantae (5)	71	**77**	73	67	73	20	43	32	66	63	51	55	49	64
Stramenopiles (1)	59	73	—	34	60	7	25	12	42	73	52	54	43	55
Choanoflagellida (1)	70	67	34	—	74	22	71	57	**78**	48	53	53	42	72
Euglenozoa (4)	**75**	73	60	74	**79**	14	55	53	73	65	54	57	47	73
Alveolata (6)	32	20	7	22	14	**96**	15	12	29	−5	−7	−4	45	6
Amoebozoa (2)	55	43	25	71	55	15	**99**	39	56	17	18	12	28	48
Diplomonadida (3)	47	32	12	57	53	12	39	**94**	58	28	31	34	35	60
Fungi (58)	**75**	66	42	**78**	73	29	56	58	**80**	44	39	43	46	66
Acidobacteria (1)	55	63	73	48	65	−5	17	28	44	—	**83**	**84**	44	70
Actinobacteria (14)	43	51	52	53	54	−7	18	31	39	**83**	**90**	**85**	32	68
Proteobacteria (8)	48	55	54	53	57	−4	12	34	43	**84**	**85**	**84**	39	70
Bacteroidetes (2)	52	49	43	42	47	45	28	35	46	44	32	39	64	54
Chloroflexi (1)	69	64	55	72	73	6	48	60	66	70	68	70	54	—

From Table 4 four clusters can be selected with a high correlation coefficient between the numbers of proteins where all considered patterns appear for all pairs between 17 animal proteomes. The first cluster corresponds to phylum Chordata (7 proteomes), the second corresponds to Arthropoda (5 proteomes), the third to Nematoda (4 proteomes), and the fourth to Cnidaria (only 1 proteome). In Tables 4 and 5, bold formatting is used to show a correlation higher than 75%, normal size of numbers to show the correlation from 50% to 75%, and smaller size of numbers to show the correlation smaller than 50%. From Table 4 it is evident that the number of proteins from the human proteome correlates with that from chicken and fish lesser than with bovine, rat, and mouse proteomes. At the same time, the correlation between the number of proteins from proteomes from the Chordata phylum is high for such proteomes as *C. briggsae and C. elegans*. High correlation coefficients also are observed for such pairs as *T. spiralis* for the Arthropoda proteomes, and *N. vectensis* for the Chordata proteomes.

Combining the motif discovery and disorder protein segment identification in the clustered PDB allows us to create the largest library of the disordered patterns. At present the library includes 141 disordered patterns. Such an approach is promising for further studying and understanding the functional role of the obtained patterns in different proteomes. We came to some general conclusions after analysis of 123 proteomes. The disordered patterns appear more often in eukaryotic than in bacterial proteomes. We can conclude that the occurrence of disordered patterns is more monotonous within the same kingdom (phylum) than between kingdoms (phyla). One can suggest that such short similar motifs are responsible for common functions for nonhomologous, unrelated proteins from different organisms.

## Supporting Information

Dataset S1 List of 141 disordered patterns.
(XLS)

Dataset S2 Number of proteins and residues for each out of 123 proteomes.
(XLS)

Dataset S3 Comparison of the new and the previous libraries of disordered patterns.
(XLS)

Dataset S4 Pairs of patterns which appear in the same protein from the whole clustered PDB.
(XLS)

Dataset S5 Occurrence of disordered patterns in 97 eukaryotic and 26 bacterial proteomes in the cases of precise and imcomplete coincidence.
(XLS)
